# Recontextualizing Dance Skills: Overcoming Impediments to Motor Learning and Expressivity in Ballet Dancers

**DOI:** 10.3389/fpsyg.2016.00431

**Published:** 2016-03-24

**Authors:** Janet Karin

**Affiliations:** ^1^Australian Ballet SchoolMelbourne, VIC, Australia; ^2^Australian Catholic UniversityMelbourne, VIC, Australia; ^3^University of CanberraCanberra, ACT, Australia

**Keywords:** neuromotor, psychology, imagery, implicit learning, naïve beliefs, ballet, motor control, dance

## Abstract

The process of transmitting ballet’s complex technique to young dancers can interfere with the innate processes that give rise to efficient, expressive and harmonious movement. With the intention of identifying possible solutions, this article draws on research across the fields of neurology, psychology, motor learning, and education, and considers their relevance to ballet as an art form, a technique, and a training methodology. The integration of dancers’ technique and expressivity is a core theme throughout the paper. A brief outline of the historical development of ballet’s aesthetics and training methods leads into factors that influence dancers’ performance. An exploration of the role of the neuromotor system in motor learning and the acquisition of expert skills reveals the roles of sensory awareness, imagery, and intention in cuing efficient, expressive movement. It also indicates potentially detrimental effects of conscious muscle control, explicit learning and persistent naïve beliefs. Finally, the paper presents a new theory regarding the acquisition of ballet skills. Recontextualization theory proposes that placing a problematic task within a new context may engender a new conceptual approach and/or sensory intention, and hence the genesis of new motor programs; and that these new programs may lead to performance that is more efficient, more rewarding for the dancer, more pleasing aesthetically, and more expressive. From an anecdotal point of view, this theory appears to be supported by the progress of many dancers at various stages of their dancing lives.

## Introduction

Dance is a natural and universal form of expression, but classical ballet has a sophisticated aesthetic and complex technique. Ballet dancers strive to make this aesthetic and technique look and feel completely spontaneous and expressive. Physical factors may limit a dancer’s skill, but various other elements can impede realization of a dancer’s technical and artistic potential. Part One of this paper outlines historical, aesthetic and training perspectives. Part Two describes research relating to common difficulties faced by dancers. Part Three presents a new theory for overcoming impediments to optimal performance.

## Background

### Historical Development

From the 17th century, ballet has exemplified the classical ideals of proportion, harmony, and mastery. Early instruction manuals (Weaver, 1706; Noverre, 1803; Blasis, 1820, 1828) made little reference to joint articulation or torso control, so we can assume that dancers achieved the desired lines and movement according to their individual physiques and preferences. Over the last century, aesthetic purity and technical virtuosity have become increasingly important. Dancers now require finer control throughout the body, with the limbs and upper body tracing clear lines around the “calm center” of the pelvis.

### Aesthetic Criteria

To the observer, ballet dancers’ bodies create pure geometric shapes, even though some of these shapes are anatomically impossible. When the dancer imagines the ideal, the observer’s mirror system (Calvo-Merino et al., 2005; Cross et al., 2006; Shamay-Tsoory, 2011; Cross and Ticini, 2012; Gentsch et al., 2016) allows the observer “to understand the action of others ‘from the inside’ and gives the observer a first-person grasp of the motor goals and intentions of other individuals” (Rizzolatti and Sinigaglia, 2010). Hence, the observer perceives the dancer’s intention rather than human limitations, making her performance aesthetically convincing.

### Contemporary Approaches to Training Dancers

Due to its complexity, ballet training is often delivered through verbal description and physical demonstration. In verbal instruction, some teachers focus on the placement and movement of body parts, for instance “straighten your knee.” Although this approach seems sound, it relies on existing motor programs that may be suboptimal.

Others describe and show muscle recruitment, as in “pull up your thigh.” Muscle instruction usually refers to contraction of the main agonist, doubtless because it is easy to see and palpate. However, conscious muscle activation counteracts the role of the neuromotor system (NMS) in creating the complex muscular coordination and response to perturbation essential for smooth, efficient movement (Carson and Riek, 2001; Hanakawa et al., 2001, 2003; Nielsen, 2004).

Some teachers say “lengthen your hamstrings,” even though the hamstrings cannot be lengthened independently. The image prompts the NMS to coordinate various other muscles to lengthen the hamstrings and so straighten the knee. Imagery of energy streaming from the hip through the leg also stimulates the NMS to straighten the knee.

## Research Relating to Potential Impediments to Dance Performance

Dancers apply instruction in the context of their pre-existing motor programs, individual beliefs, and learning approach. Current research illuminates these and other factors that can impede or facilitate optimal performance, so forming a basis for the strategies presented in part 3.

### Motor Control

Although current theories on the initiation and execution of voluntary movement differ in detail, it is generally considered that the NMS coordinates sensory information and conscious intention to formulate and monitor motor programs in order to produce the intended movement (Lau et al., 2004; Haggard, 2005, 2009; Jensen et al., 2014; Grüneberg et al., 2015; Gentsch et al., 2016). Each motor program coordinates a variety of neural impulses that signal specific muscles to behave in certain ways to achieve a goal, and these impulses are continually modified in response to feedback from the sensory system (Ioffe, 2004; Pacherie, 2008; Koziol et al., 2012). Even when movement appears to be limited to a few body parts, the NMS is involved in moving, supporting, and stabilizing the whole body and responding to breathing and postural sway (Lam et al., 2009; Masters and Maxwell, 2004; Miller et al., 2010). Each person develops his own repertoire of motor programs according to his daily experiences and physical capabilities. Regular use strengthens the relationship between these motor programs and their goal, and they become the basis for learning further movement skills (Criscimagna-Hemminger and Shadmehr, 2008; Kantak et al., 2012; Scheidt et al., 2012).

As ballet instruction often focuses on muscle recruitment, dancers frequently oppose any inaccuracies with muscular force. The resulting conflict between habitual and new motor actions creates inefficient tension, precluding natural coordination and the formation of more efficient motor programs, and increasing injury risk. Further explicit instruction exacerbates the problem. If the dancer’s conceptual understanding of the task changes, motor imagery can activate the different non-conscious processes involved in learning new motor tasks (Jackson et al., 2001).

### Motor Imagery (MI)

The literature cites many forms of mental imagery, including movement imagery, mental practice, imagery rehearsal, visualization, kinaesthetic imagery, visuomotor behavioral rehearsal and internal imagery (Lotze and Halsband, 2006; Guillot and Collet, 2008; McAvinue and Robertson, 2008). Imagery is often effective in improving performance (Ranganathan et al., 2004; Smith et al., 2007, 2008; Lebon et al., 2010), although exceptions are found in certain types, time, duration, and other conditions and with negative imagery (Beilock et al., 2001; Weinberg, 2008; Kingsley et al., 2013).

MI is frequently defined as the mental rehearsal of movements without actual execution (Solodkin et al., 2004; Anema and Dijkerman, 2013). MI activates most of the areas that will execute the specific movement (Ehrsson et al., 2003), including motor programming (Naito et al., 2002). In contrast to imagery of the movement itself, metaphorical MI presents other shapes, movements and ideas that may enhance motor performance (Short et al., 2001).

Kinaesthetic MI involves “feeling” the sensations the imagined movement would produce (Naito et al., 2002). They may be realistic sensations such as “hinging” or metaphorical sensations like “evaporating”. Kinaesthetic images rely on sensory awareness and an internal representation of the body (Longo et al., 2009). Body representation can be changed through visual and tactile stimuli (Ehrsson et al., 2005; Moseley, 2008; Rothgangel et al., 2011; Walsh et al., 2011), and sustained practice (Classen et al., 1998).

Visual MI entails mentally “seeing” a shape or movement. Internal visual imagery is a mental picture of yourself. External visual imagery is a mental picture of another person or your image in the mirror (Guillot et al., 2009; Heremans et al., 2009). Internal imagery produces stronger motor activation than external imagery (Jackson et al., 2006).

Visual and kinaesthetic imagery “are mediated through separate neural systems, which contribute differently during processes of motor learning” (Guillot et al., 2009). Kinaesthetic imagery bears a closer resemblance to actual movement execution and even influences heart and respiratory rates, possibly as preparation for actual movement (Solodkin et al., 2004). The NMS responds to kinaesthetic and visual imagery by retrieving motor memories of similar movements (Nyberg et al., 2001; Naito et al., 2002).

Metaphorical imagery can stimulate sensory, kinaesthetic, emotional and aesthetic responses. “A dart” may assist speed and lightness. “Feet sinking through the floor” may improve foot alignment and proprioception, and may also help the dancer feel emotionally “grounded.” “Seeing with the back of your body” may evoke motor responses that are difficult to convey technically. Ballet’s fundamental aesthetic of mastery may be inspired by the metaphor of sunlight radiating from the body in every direction. The kinaesthetic cues embedded in metaphors may assist the NMS in improving motor skill (Solodkin et al., 2004). As a form of imagery, metaphorical thinking “provides a cognitive and emotional mode of communication between choreographer, performer, and observer” (Stevens et al., 2003). Imagery can encapsulate the dancer’s intention, sensory experience, and emotion, and these are received by the observer’s mirror system (Hayes et al., 2008).

In ballet training, the body is often manipulated consciously, with each part arranged as directed to produce a facsimile of the technique’s shapes and movements. Conversely, metaphor, analogy, and imagery are direct cues to the NMS, promoting physical ease and artistic richness and facilitating implicit learning (Liao and Masters, 2001).

### Implicit and Explicit Learning

Learning in dance can occur implicitly, in response to imagery and sensory cues, or explicitly, through conscious verbal-analytical processing of a movement’s rules or mechanics. Implicit and explicit learning employ partly distinct neural circuitry, with implicit learning systems relying on phylogenetically older processes (Karabanov et al., 2010). In learning very complex skills, dancers may benefit by “hybrid learning,” which ideally combines the benefits of implicit and explicit processes (Raab, 2015).

Explicit learning increases motor performance through rules and feedback, and is dominated by declarative forms of control (Masters and Maxwell, 2004; Lam et al., 2009). Explicit learning involves conscious recollection of each element and its order in the sequence (Kantak et al., 2012). Since explicit processes depend on working memory, they are vulnerable to disruption by stressors or fatigue (Lam et al., 2009).

Implicit motor learning is a “background subconscious mental subroutine establishing the correct combination, timing, or magnitude of activation of the muscle” (Matsumura et al., 2004). Most simple skills are acquired implicitly, with the NMS selecting the most efficient way to achieve the goal, thereby maximizing facility and minimizing energy expenditure (Bläsing et al., 2012). Implicitly learned skills require less working memory and are robust against disruption (Karabanov et al., 2009; Zhu et al., 2011). When asked to describe an implicitly learned skill, people’s responses are usually delayed while they translate implicit knowledge into verbal form.

Implicit motor learning techniques circumvent effortful, cognitive stages that are typical of unskilled performers, thereby promoting more expert-like performance (Masters and Maxwell, 2004; Masters et al., 2008). Implicit processes should dominate at the expert level, although explicit learning can become implicit with practice (Lam et al., 2009). In situations of high pressure, experts sometimes find their normally high-level skills become confused and inefficient. Maxwell et al. (2006) and Masters and Maxwell (2008) suggest this is due to reinvestment, where rules acquired during explicit learning interrupt implicitly controlled skills. An early and continued focus on implicit learning may protect the performer against reinvestment (Masters and Maxwell, 2004).

Dancers usually receive extensive explicit instruction throughout their training, and are constantly asked to verbalize their learning. Given the difficulty of resisting their strong explicit knowledge, dancers could benefit by placing the task in a completely different context and so “learning a new movement.”

### Beliefs and Movement

From their earliest years, children acquire naïve beliefs to make sense of their experiences. A child wears a “warm coat” in winter. Since the “warm coat” makes him warm, he believes that it generates heat. Similarly, young children believe that the sun revolves around the earth and whales are fish (Shtulman and Valcarcel, 2012). Later, scientific facts challenge naive beliefs, leading to the formation of new “learned” beliefs. However, the naïve beliefs remain. When there is no conflict between beliefs, people employ fast, unconscious processes to make decisions. When naïve and learned beliefs differ, the “right” belief must be selected through cognitive processing, resulting in slower responses and less accuracy under pressure (Osman and Stavy, 2006; Stavy and Babai, 2010). Stress, fatigue and cognitive pressure encourage reversion to naïve beliefs (Kelemen and Rosset, 2009; Shtulman and Valcarcel, 2012; Kelemen et al., 2013).

I have observed that dancers’ motor programs can be strongly affected by naïve childhood beliefs. Frequent reference to “pointed toes” promotes the belief that toes should curl under. Remarks about “dancers’ lovely straight backs” encourage the belief that dancers have rigid spines. Praise for “working hard” convinces children that muscle tension is key to success. Consequently, children willingly acquire motor programs that correspond with their inaccurate naïve beliefs.

Training should ensure the development of beneficial beliefs and motor programs. However, the NMS still supports the motor programs allied with the naïve beliefs, ready to take over when the dancer is fatigued, stressed or under cognitive pressure. Fatigued and stressed dancers are particularly vulnerable to injury when the motor programs attached to a counterproductive naïve belief unexpectedly interfere with their seemingly well-established motor control.

Several strategies have been proposed to overcome conflict between naïve and learned beliefs (Guzzetti, 2000; Baxter et al., 2004; Babai et al., 2010). These approaches may help correct errors, but they do not appear to influence strongly established motor programs. Ohlsson (2009) proposed transferring a concept from another domain or context to the problem situation, but I propose transferring the problematic task to another context.

### Expressivity

From birth, we are adept at expressing sadness, contentment and anger, conveying our emotions through contracted muscles, restricted breathing, sweating, restlessness or tears. The mirror system activates “common circuits when observing sensations or emotions felt by others, and when experiencing these sensations and emotions ourselves” (Bastiaansen et al., 2009). “Understanding the current state, future intentions, and emotions of another person via observing them is crucial for social interactions” (Hayes et al., 2008). The source of empathetic experience in dance is fundamentally somatic, and bodily empathy allows observers to feel the dancer’s expressive intent (Warburton, 2011; Sevdalis and Raab, 2014).

Conversely, the body and movement can influence emotions. Various studies show that manipulating subjects’ facial expressions, body posture, and head movements can influence their emotional state (Niedenthal, 2007). In class, dancers are positively affected by exuberant exercises. Expressive choreography stimulates dancers’ emotions, enabling them to convey the choreographer’s meaning to others. By contrast, focusing on movement mechanics is unlikely to activate the brain regions that convey emotion to the observer (Bastiaansen et al., 2009).

Inevitably, ballet teachers give verbal instruction based on their own experience, so students receive knowledge through the prism of motor programs appropriate for their teacher’s individual physicality. When demonstrating movements, teachers also transmit their own cultural background, personality, musicality and artistic sensibility. Replicating the teacher’s movement style and expression may restrict development of the young dancer’s unique potential.

Young dancers attempting to reproduce a sophisticated style of movement designed for adult dancers frequently subjugate their natural expressivity in favor of stereotypical expression. This can disconnect their true emotional life from their technique. By rediscovering technical movements in a different context, the natural fusion of emotion and movement can gradually be restored.

## Recontextualization Theory: Placing Tasks in New Contexts to Enhance Performance

Conversations with exceptionally successful dancers reveal a common theme—they trust imagery and their sensory systems to produce dance. For most of her training and professional career as a Principal dancer, Kirsty Martin (2015, personal communication) focused on imagery rather than technical rules, and on feeling balanced, calm, and relaxed. After a period of explicit coaching at the height of her career, her dancing felt awkward and unenjoyable. She regained ease and expressivity by refocusing on imagery and “the feeling of dancing.” After long experience as a teacher specializing in dancers’ underperformance, I conclude that persistent difficulties are usually related to the context within which the dancer sees the task. A jump may be seen in the context of conscious muscle activation. In an imagery context, the dancer may embody a bounding kangaroo. In a sensory context, the jump may “be” a beach ball that has been pushed under the water and then released. Each context will cue the NMS differently, and the outcome will reflect the context.

Words have different meanings according to their context. In the kitchen, the meaning of the word “tap” is related to water. In the dance world, “tap” may mean Fred Astaire or Gregory Hines. Different meanings are attached to a tap at the door, on a keyboard, with a hammer, and into a telephone line. I theorize that placing problematic skills within different contexts prompts the NMS to identify them as new skills. In particular, sensory and/or imagery contexts may lead to more effective motor programs and consequently better performance and expressive ability. Often, sensory and imagery cues are integrated to strengthen multimodal processing.

### Cueing within a Sensory Context

Ideally, all voluntary movement is guided by information from the sensory system (Dijkerman and de Haan, 2007).

Explicit instruction can align bones in simple postures, but dancers need efficient alignment throughout all positions and movements. By placing alignment in a sensory context, focused awareness of the back half of the body improves body representation, prompts the NMS to align the body efficiently when still and moving, improves balance and increases expressivity.

Breathing, emotions and movement are normally strongly interwoven, with each influencing the others. Young dancers often hold their breath, interfering with their movement’s integrity and precluding expressivity. In a sensory context, exploring various relationships between breathing and moving may enhance expressive potential and release physical and psychological tension (Brodie and Lobel, 2012).

Some dancers respond to challenge by “pulling up,” or generally engaging their muscles. This often leads to breath-holding, inefficient muscle tension and instability. In a sensory context, dancers may allow their weight to melt through the floor, increasing stability and a sense of security.

Sensory images such as moving underwater, skiing into the wind, melting into the floor, or swooping down a roller coaster may awaken expressive responses. Limitless combinations of breathing, music, movement and spatial awareness can give the dancer a richly expressive vocabulary.

### Cueing within an Imagery Context

Kinaesthetic imagery provides the NMS with new cues for optimal performance (Jackson et al., 2001).

Kinaesthetic imagery of a fluid central line flowing through the major weight-bearing joints while adapting to the position or movement cues the NMS to activate the stabilizing muscles and maintain safe function.

For some dancers, “arabesque” means forceful co-contraction of the back and abdominal muscles, preventing natural coordination and increasing risk of injury. In an imagery context, “arabesque” may be a crescent moon, a swoosh, or a floating bowl, reconceptualising arabesque as an embodiment of harmony and beauty.

A naïve belief in balance as total stillness can interfere with natural proprioception and postural sway. In an imagery context, balancing can be reconceptualised as being “suspended in space by spider-webs.”

In traveling steps, explicit control resists unwanted pelvic movement with conscious muscular effort. In an imagery context, the pelvis may glide smoothly along shiny steel rails, prompting the NMS to program effortless control.

## Conclusion

I theorize that each dancer contextualizes each task differently, and this context influences the way the NMS programs the movement. If the NMS fails to produce the desired result, I propose that placing the task in a different context may facilitate optimal performance and unleash the dancer’s potential.

## Author Contributions

The author confirms being the sole contributor of this work and approved it for publication.

## Conflict of Interest Statement

The author declares that the research was conducted in the absence of any commercial or financial relationships that could be construed as a potential conflict of interest.
